# Fabrication of a Textile-Based Wearable Blood Leakage Sensor Using Screen-Offset Printing

**DOI:** 10.3390/s18010240

**Published:** 2018-01-15

**Authors:** Ken-ichi Nomura, Yoshinori Horii, Shusuke Kanazawa, Yasuyuki Kusaka, Hirobumi Ushijima

**Affiliations:** Flexible Electronics Research Center, National Institute of Advanced Industrial Science and Technology (AIST), 1-1-1 Higashi, Tsukuba 305-8565, Japan; horii-y@aist.go.jp (Y.H.); kanazawa-s@aist.go.jp (S.K.); y-kusaka@aist.go.jp (Y.K.); h-ushijima@aist.go.jp (H.U.)

**Keywords:** blood leakage, flexible electronics, impedance, printed electronics, screen printing, screen-offset printing, wearable devices

## Abstract

We fabricate a wearable blood leakage sensor on a cotton textile by combining two newly developed techniques. First, we employ a screen-offset printing technique that avoids blurring, short circuiting between adjacent conductive patterns, and electrode fracturing to form an interdigitated electrode structure for the sensor on a textile. Furthermore, we develop a scheme to distinguish blood from other substances by utilizing the specific dielectric dispersion of blood observed in the sub-megahertz frequency range. The sensor can detect blood volumes as low as 15 μL, which is significantly lower than those of commercially available products (which can detect approximately 1 mL of blood) and comparable to a recently reported value of approximately 10 μL. In this study, we merge two technologies to develop a more practical skin-friendly sensor that can be applied for safe, stress-free blood leakage monitoring during hemodialysis.

## 1. Introduction

Three million patients worldwide need renal replacement therapy [1]. Hemodialysis is an effective renal replacement therapy in which human blood is exsanguinated via a needle inserted into the patient’s arm, external equipment purifies the blood, and the blood returns to the patient’s body via another needle. During hemodialysis, severe bleeding occurs if the needle is pulled out of the arm, which may be life-threatening. However, doctors and nurses cannot constantly check whether bleeding occurs, since it takes a few hours to complete each hemodialysis.

To prevent such accidents during hemodialysis, various sensors to detect blood leakage have been proposed [2,3,4,5,6,7]. Of these, a flexible sensor based on a film substrate fabricated using printing technology seems appropriate [6,7]. Patients must be dialyzed two or three times per week; therefore, mass-production techniques must be applied to provide many disposable sensors inexpensively. However, a film substrate is unsuitable for attaching to the arm, as patients will feel some discomfort because plastic film is hard and essentially impermeable to air. Hence, it would be preferable to develop a sensing structure on a soft, breathable material, such as a textile. Another problem in sensing blood leakage is low accuracy. For example, a sensor that detects conductivity between two electrodes detects blood via an increase in conductivity because the electrodes are covered by blood. However, this type of sensor detects other liquid materials such as sweat, which is also conductive. Patients may sweat due to fear or pain caused by inserting needles, or because of the temperature of the treatment room. 

**Figure 1:**
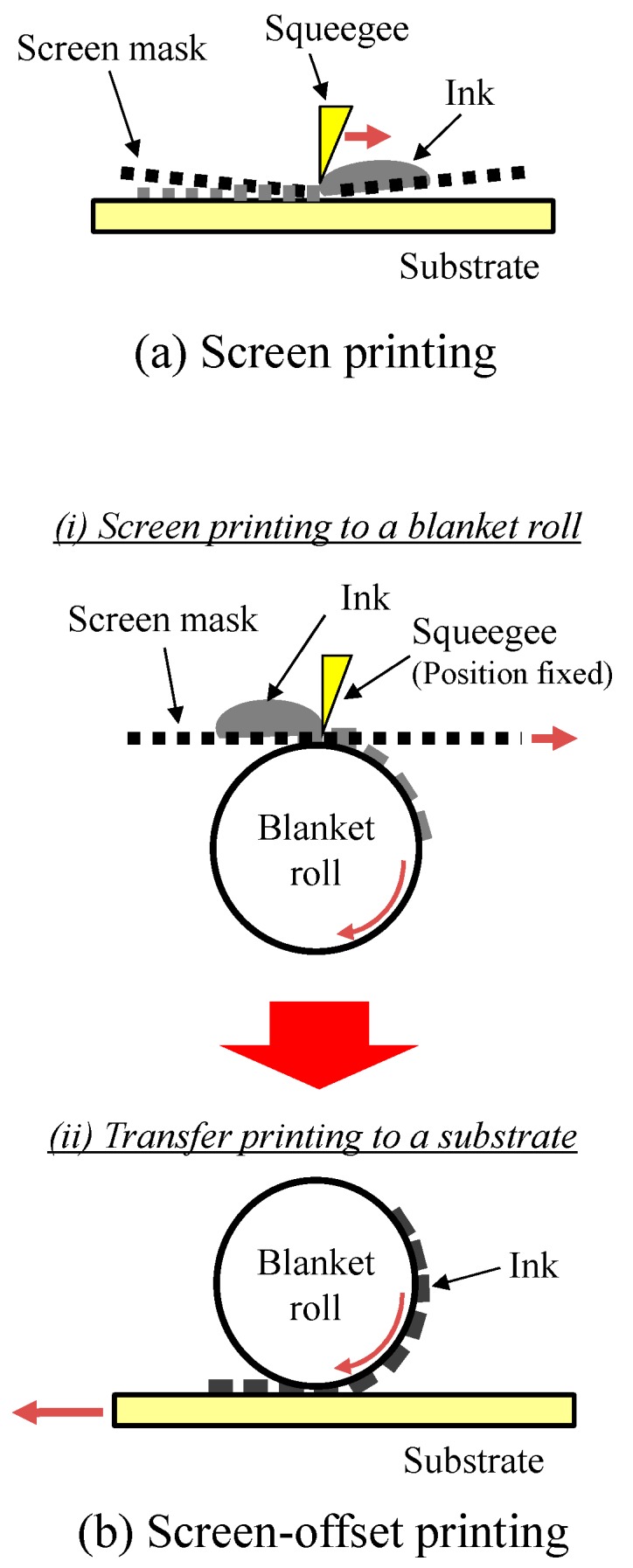
Schematic images of (**a**) screen printing and (**b**) screen-offset printing.

To address these issues, we combine two technologies. The first is a simple formation of conductive patterns on textiles by applying a screen-offset printing technique that we developed [8,9,10,11]. Printing is very useful technique, since it enables rapid pattern formation at low cost. Flexographic printing [12,13,14], microcontact printing [15], gravure printing [16], gravure-offset printing [17,18], soft blanket gravure printing [19], reverse-offset printing [20,21,22,23], and adhesion contrast planography [24] have all been proposed as pattern formation techniques. Of these, we employ screen-offset printing, which is based on screen printing. Screen printing is a technique that uses a mask composed of a mesh and patterned emulsion; conductive ink is patterned on a substrate through holes on the mask, as shown in Figure 1a. Screen printing is widely used because of its low printing error, inexpensive machines and masks, and simplicity compared to other printing methods. Conductive patterns of various electronic components have been fabricated using screen printing [25,26,27,28,29]; however, it is difficult to apply conventional screen printing to textiles in some cases, since fluid ink tends to permeate the textile, resulting in blurring. Ag ink was screen-printed on cotton fabrics and functioned as conductive wires [30]. However, the reported wire width was approximately 1 mm; fine patterns with widths less than 1 mm would be difficult to form due to ink bleeding, which limits its range of application. 

Therefore, we apply an improved screen printing process called screen-offset printing to properly form sensor electrodes on textiles. Figure 1b shows a schematic of the roll-blanket screen-offset printing method used in this study. In this method, conductive ink is first screen-printed on a silicone blanket; then, the ink patterns formed on the blanket are transferred to the final substrate. One important aspect of this method is that the silicone blanket absorbs organic solvents in the ink [31]. This absorption increases the ink’s viscosity on the blanket, preventing blurring and enabling the production of fine printing patterns. Such high-viscosity conductive inks can function as a physically floating cantilever and bridge without any cracks during the transfer processes [32,33]. Thus, screen-offset printing can easily form conductive patterns on a highly porous textile-based material by bridging the pores in the material to print conductive patterns less than 1 mm wide.

The second method we established distinguishes blood from other substances using interdigitated conductive patterns formed on textiles; we can detect whether blood permeates the textile by observing the frequency dependence of electrical parameters between the interdigitated electrodes. To our knowledge, this is the first report of such a fabrication method or its application to blood leakage sensors. It is also the first practical blood leakage sensor; other candidates are flawed, as shown in Table 1. Our newly developed sensor may open the way to realizing safe hemodialysis. 

## 2. Materials and Methods 

### 2.1. Formation of Interdigitated Electrodes on Textiles

To form electrodes on textiles, we employed a screen-offset printing machine (SO-1010) manufactured by the Mino Group, Gujo, Japan. Ag ink (MP-501SO/6322O), which can be used as room-temperature curable conductive ink, was also acquired from the Mino Group. The ink was overprinted two or three times on the silicone blanket to prevent wiring disconnection because of an overly thin conductive pattern. A screen mask with an interdigitated pattern was purchased from Mesh Corp., Osaka, Japan. Silicone blankets were composed of a polydimethylsiloxane sheet and a base film. Substrates used were commercially available cotton textiles. Using these materials, we printed interdigitated electrode patterns for a blood leakage sensor with line and space (L/S) widths of 500 μm/1000 μm on the textile.

### 2.2. Detection of Blood Permeating the Textile

We purchased a human whole blood sample, in which EDTA-2K was added as an anticoagulant, from Tennessee Blood Service via Bizcom Japan, and phosphate-buffered saline (PBS) was used as a substitute for sweat. Before and after dropping blood or PBS onto the textile-based sensor, the impedance *Z* and phase *θ* between the electrodes were measured using a 3532-50 LCR HiTESTER manufactured by Hioki E. E. From the obtained data, the reactance *X* was calculated as

(1) X=Zsinθ [Ω].

In this study, we will discuss the frequency dependence of the values of

(2) $$-\frac{1}{\omega X}=-\frac{1}{2\pi fX}\;\left[s/\Omega \right],$$

where *ω* is angular frequency and *f* is frequency. *X* can thus be represented as

(3) $$X=\omega L-\frac{1}{\omega C}=2\pi fL-\frac{1}{2\pi fC}\;\left[\Omega \right],$$

where *L* and *C* are the nominal inductive and capacitive lumped components, respectively, in a simple (series connected) RLC model. Thus, the value indicated in Equation (2) is equivalent to *C*; namely,

(4) $$C=-\frac{1}{\omega X}\;\left[F\right]$$

if the sensor contains no inductive component. The value of *C* is often discussed when dealing with dielectric dispersion assuming that *L* = 0. However, the value estimated in the experiment is *X*, and we cannot determine accurate values for *L* and *C*. Therefore, in this study, we discuss the value indicated in Equation (2).

## 3. Results and Discussion

**Figure 2:**
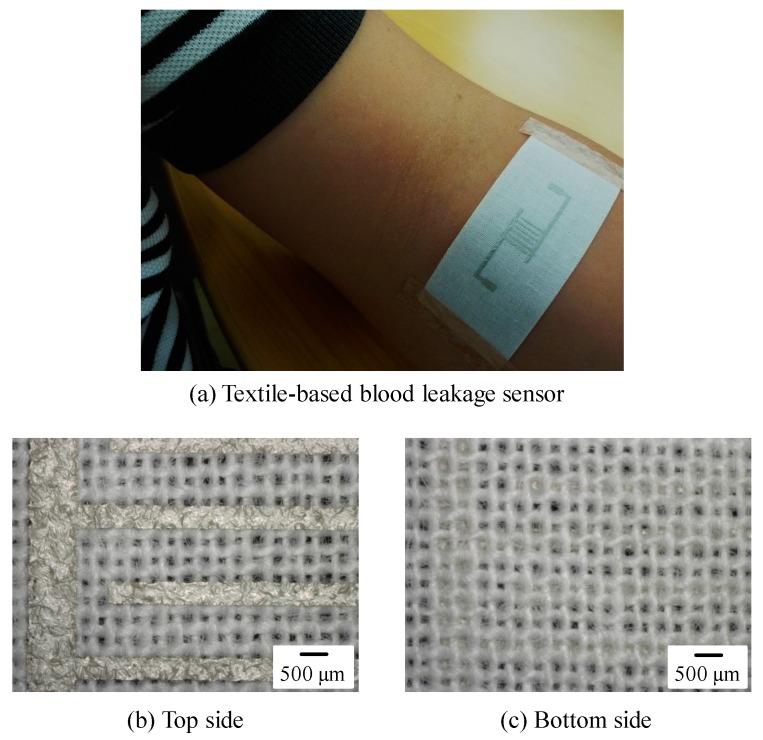
(**a**) Textile-based blood leakage sensor, and microscopy images of its (**b**) top and (**c**) bottom.

**Figure 3:**
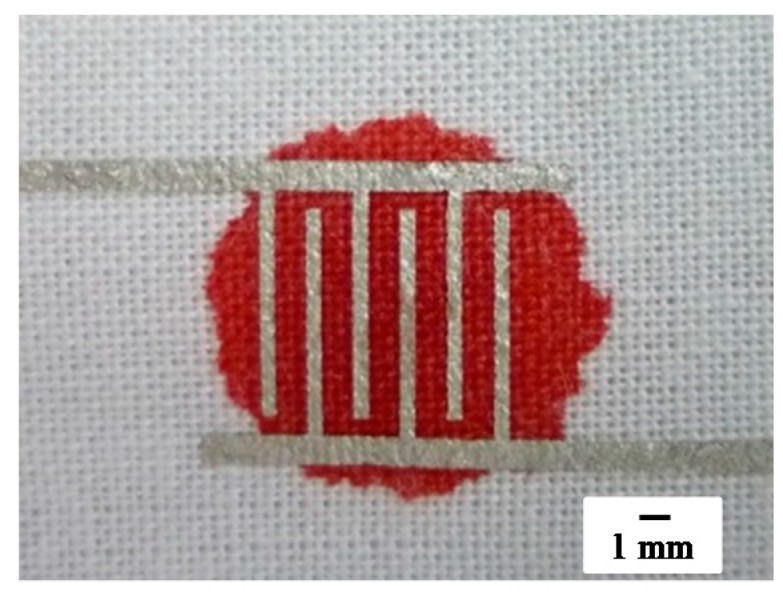
Photograph of the sensor after dropping 15 μL of blood.

**Figure 4:**
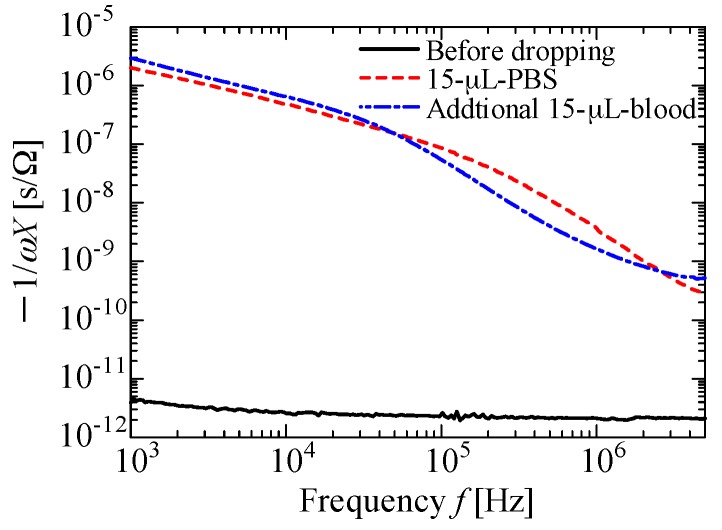
Sensor values of −1/*ωX* as a function of frequency *f* obtained before and after dropping 15 μL of PBS, and after dropping an additional 15 μL of blood.

Figure 2a shows a photograph of an arm of one of the authors with the developed textile-based sensor attached, and Figure 2b shows a microscopy image of the sensor. The electrode patterns maintained the originally designed width, and the patterns bridge the pores in the textile. We also confirmed that the printed patterns on the textile were conductive. Figure 2c shows the reverse side of the textile. Screen-offset printing clearly prevents ink from permeating the textile. Note that printed electrodes maintained their conductivity even if the ink was covered with PBS for 5 h. Also note that the sensor shown in Figure 2a was just attached to the arm, and all the experiments described in the present paper were performed without using human bodies. Next, using our textile-based sensor, we measured electrical properties under various conditions; blood or PBS was dropped on the interdigitated pattern-formed textile. Figure 3 shows a photograph of 15 μL of blood dropped on the textile. The blood permeated and spread in the textile. Figure 4 shows a plot of −1/*ωX* as a function of frequency *f* obtained after 15 μL of PBS was dropped on the textile, and then an additional 15 μL of blood was dropped. Note that the measurement was performed in a frequency range of 1 kHz–5 MHz. In this range, 200 points were measured for one droplet condition; it took 2 to 3 min to complete the scan from 1 kHz to 5 MHz. After finishing one measurement, we immediately dropped additional liquid and conducted the next measurement. As shown in Figure 4, the slope at a frequency of approximately 100 kHz differed between PBS and additional blood droplet cases. This change in spectra seems to be due to dielectric relaxation of erythrocytes in the blood [34,35], indicating that we can easily distinguish blood from other substances by comparing these slope values. 

**Figure 5:**
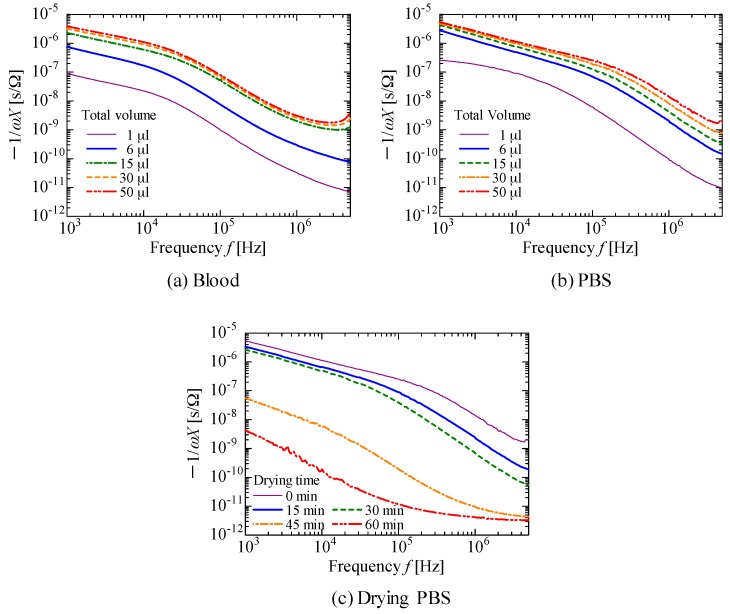
Sensor values of −1/*ωX* as a function of frequency *f* obtained when up to 50 μL of (**a**) blood or (**b**) PBS was dropped on the sensor. The sensor, on which a total volume of 50 μL of PBS was dropped, was dried at room temperature; spectral dependence on drying time is shown in (**c**).

**Figure 6:**
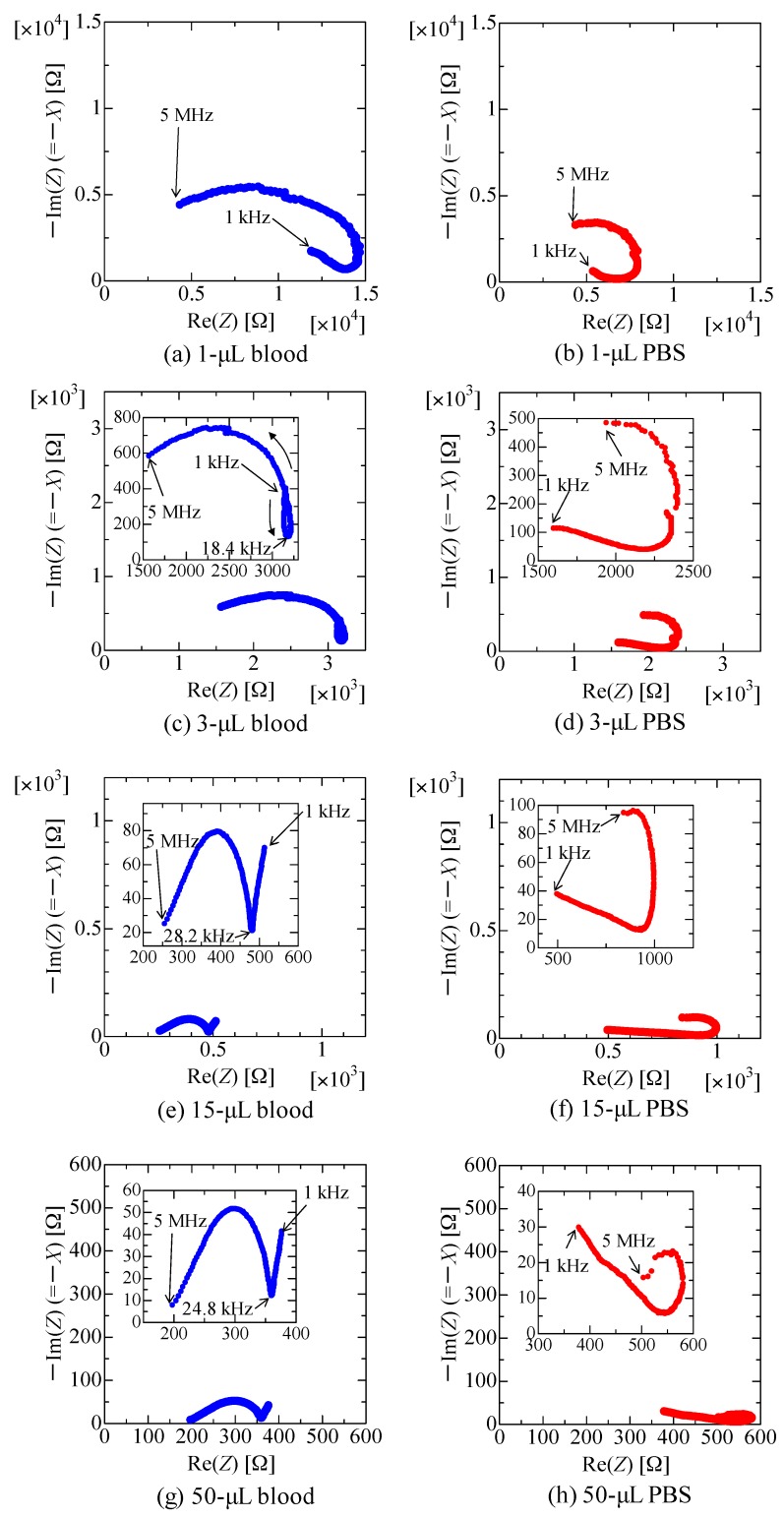
Nyquist plots of the sensors under representative conditions; after dropping 1 μL of (**a**) blood or (**b**) PBS; 3 μL of (**c**) blood or (**d**) PBS; 15 μL of (**e**) blood or (**f**) PBS; and 50 μL of (**g**) blood or (**h**) PBS. Insets are enlarged figures.

Figure 5a shows the spectra obtained when up to 50 μL of blood was dropped on the sensor, whereas Figure 5b shows the spectra obtained when PBS was dropped. Figure 5c shows the spectra obtained when drying the 50 μL of PBS dropped on sensor shown in Figure 5b at room temperature; although blood leakage caused by needles being pulled out should occur continuously, sweat may stop and the textile may dry. Note that, for clarity, representative data of the measured values are shown in Figure 5a‒c. Furthermore, note that data for the 50 μL droplet shown in Figure 5b is the same as the initial data in Figure 5c. In addition, to confirm the dielectric relaxation of the blood, Figure 6a,c,e,g show Nyquist plots of the sensors after dropping total volumes of 1, 3, 15, and 50 μL of blood, respectively. The corresponding figures using PBS are shown in Figure 6b,d,f,h. The plot shape in the case of 1 μL of blood, shown in Figure 6a, is similar to that in the cases of 1 μL of PBS (Figure 6b); it is difficult to distinguish between them. However, the loop opening, which indicates dielectric relaxation, starts to appear when 3 μL of blood was dropped (Figure 6c), and it is clearly seen in the cases of 15 and 50 μL of blood (Figure 6e,g). On the other hand, such a loop opening is not observed when PBS was dropped (Figure 6b,d,f,h). These results imply that the sensor has the potential to detect as low as ~3 μL of blood by analyzing Nyquist plots. 

However, Nyquist plots require a series of resistance and reactance data; as a result, the measurement instrument becomes complex and expensive, and the time taken to complete the measurements is long. For practical use, it is better to reduce the number of parameters for simplifying the measurement process and for realizing a low-cost system; for example, eventually, the use of a cheap IC for measuring only capacitance *C* appears ideal. From this viewpoint, we attempt to establish a method that can detect blood just from the data of −1/*ωX* (≈*C*).

Figure 6e,g indicate that blood exhibits dielectric relaxation at frequencies higher than ~25 kHz, whereas it cannot be observed in the case of PBS. When dielectric relaxation occurs, capacitance *C* (≈−1/*ωX*) drastically changes [35]; the decrease in −1/*ωX* observed at a frequency of 30 kHz−1 MHz shown in Figure 5a is due to dielectric relaxation of the blood. In contrast, PBS does not demonstrate dielectric relaxation (Figure 6b,d,f,h) in the measured frequency range, indicating that a change in −1/*ωX* does not occur. However, a sensor on which small volumes of PBS (e.g., 1 μL) were dropped, shown in Figure 5b, and a sensor that was dried for 30 min, shown in Figure 5c, resulted in a significant decrease at the same frequencies; although this significant decrease in −1/*ωX* is not known, it may result in detection errors if we try to detect blood only by observing the value of the slope. 

**Figure 7:**
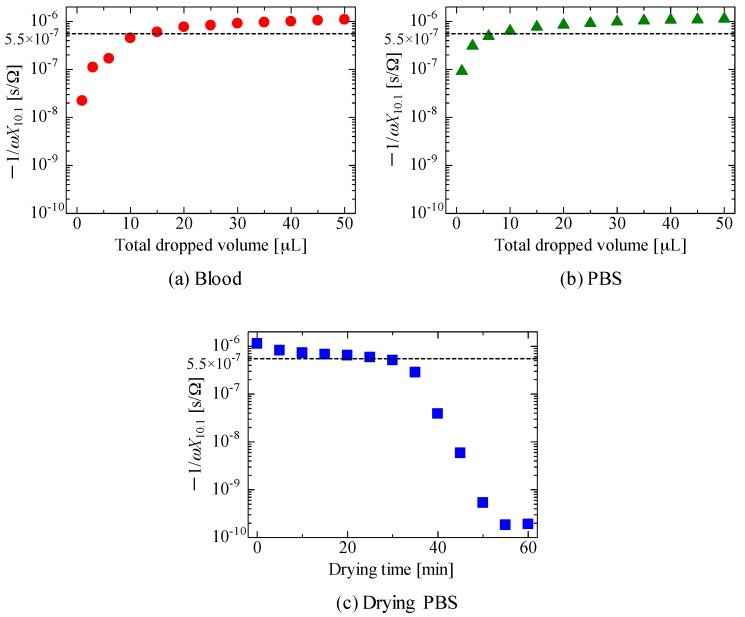
Values of −1/*ωX*_10.1_ of the sensor as a function of the total volume of (**a**) blood or (**b**) PBS dropped. The dependence of the −1/*ωX*_10.1_ of the sensor in which a total volume of 50 μL of PBS was dropped on the drying time is shown in (**c**). The assumed threshold value (5.5 × 10^−7^ s/Ω) is indicated as a visual guide.

This problem can be avoided with the following two steps. (i) At a certain frequency, an appropriate threshold of −1/*ωX* is determined; a low value is excluded at this phase. As described above, a small volume of PBS induces a large decrease in −1/*ωX* at 30 kHz−1 MHz, as shown in Figure 5b,c. Therefore, by setting a proper threshold, we extracted a larger amount of blood or PBS. (ii) Then, for the extracted conditions, we checked the decrease in −1/*ωX* caused by the dielectric relaxation of the blood. As an example of the first step, Figure 7a,b show the values of −1/*ωX*_10.1_ replotted as a function of the dropped volume corresponding to Figure 5a,b, respectively, where *X*_10.1_ is the reactance at 10.1 kHz. Similarly, Figure 7c depicts −1/*ωX*_10.1_ as a function of the drying time corresponding to Figure 5c. For example, the threshold of (i) is assumed to be −1/*ωX*_10.1_ = 5.5 × 10^−7^ [s/Ω]; cases in which the total dropped volume is less than or equal to 10 μL in Figure 7a and less than or equal to 6 μL in Figure 7b, and in which the drying time is greater than or equal to 30 min in Figure 7c are excluded at this phase.

**Figure 8:**
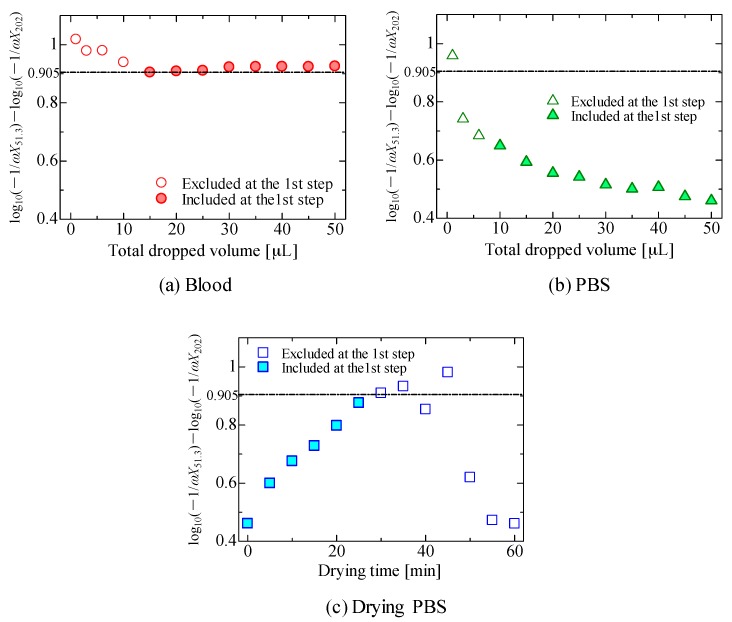
Slope values of log_10_(−1/*ωX*_51.3_) − log_10_(−1/*ωX*_202_), as a function of the volume obtained when (**a**) blood or (**b**) PBS was dropped to the sensor. The slope value as a function of the sensor’s drying time in which a total volume of 50 μL of PBS was dropped is shown in (**c**). Excluded and included conditions at the first selection step, estimated from Figure 7, are indicated in each figure. Furthermore, the minimum slope value shown in (**a**) is 0.905, which is indicated in other figures as a visual guide.

Next, we explain details of the second step. Figure 8a‒c show plots subtracting the value of −1/ωX at 202 kHz from that at 51.3 kHz, which corresponds to slope values between 51.3 and 202 kHz. Note that the *y* axis of Figure 5 is displayed logarithmically; the values of log_10_(−1/*ωX*_51.3_) − log_10_(−1/*ωX*_202_) are shown in Figure 8, where *X*_51.3_ and *X*_202_ are the reactance values at frequencies of 51.3 kHz and 202 kHz, respectively. As shown in Figure 8, the minimum slope value shown in Figure 8a, 0.905, is larger than the values obtained under other conditions shown in Figure 8b,c if we compare the values not excluded in the first step. These results indicate that blood can be distinguished from other substances. Furthermore, the detection limit of blood volume is as low as 15 μL, which is much lower than that of commercially available products (approximately 1 mL) [4], and comparable to the 10 μL reported recently [4].

Our textile-based blood leakage sensor can be fabricated simply using screen-offset printing, and can easily detect whether a small volume of blood permeates the textile. This sensor can contribute to safe hemodialysis without discomfort, and related technology is expected to create a new paradigm, an ‘Internet of Textiles’, in the near future; the experiments described in the present paper demonstrate this technique’s feasibility; we will perform practical experiments, namely, blood leakage measurements after a needle is inserted in a patient, as a next step. Furthermore, we have demonstrated that a textile-based substrate is suitable for use on human skin as a wearable device. For example, a film-based sensor can detect glucose in tears [36]. We can, in principle, detect the amount of glucose just by wiping tears using a handkerchief with sensor electrodes. Conductive materials formed on a fabric mask can serve as a sensor to detect various gases, e.g., ethanol [37], in breath. Such a new device could contribute to the early detection of disease and simple diagnostics. We will explore new biomedical and related applications using our developed technology.

## 4. Conclusions

In this study, we printed interdigitated conductive patterns on a textile material and implemented this structure as a blood leakage sensor. The screen-offset printing technique easily forms dried conductive-ink patterns on a cotton textile material. Furthermore, the printed interdigitated electrode can detect blood or PBS by extracting the case in which the value of −1/*ωX* at a certain frequency is larger than a predetermined value, and by measuring the slope of log_10_(−1/*ωX*) at sub-megahertz frequencies; dielectric relaxation of erythrocytes in the blood resulted in larger slope changes; the limit of detection for the sensor is as low as 15 μL in practice. Further, the detection limit seems to be improved to ~3 μL if we can consider the Nyquist plot. This breathable skin-friendly sensor can contribute to significantly safer hemodialysis; moreover, its low-cost fabrication using screen-offset printing can drive the widespread application of this technology.

## 5. Patent

K.N., Y.H., S.K., and H.U. are declared as inventors on a patent application (Japanese Patent Application no. 2017-184939). Items relating to the sensor in this study have yet to be commercialized.

## Figures and Tables

**Table 1:** Comparison of blood leakage sensor candidates

Type	General Feature	General Problems	Other Information
Electrical sensor (Conductivity/Impedance change)	Simple structure, cheap equipment	Low accuracy, impermeable to air	Limit of detection: 10 μL^−1^ mL [4]
Optical sensor	High accuracy	Impermeable to air, causes physical discomfort	Sensitivity: 4 × 10^−6^ blood concentration in volume [2]
Camera (visual and IR)	High accuracy	Psychologically uncomfortable	
Pressure-sensitive sheet	Simple structure, cheap equipment	Impermeable to air	
Blood-flow rate sensor	Simple structure, cheap equipment	Delayed detection	
